# Retrospective analysis of the clinical utility of blood cultures taken surrounding intensive care admission

**DOI:** 10.1186/cc13552

**Published:** 2014-03-17

**Authors:** V Humphrey, M Clark

**Affiliations:** 1 Victoria Hospital, Kirkcaldy, UK

## Introduction

This study aimed to establish clinical utility of blood cultures taken surrounding ICU admission. Blood culture cost in terms of resources and false results is well established and unnecessary cultures can cause patient harm.

## Methods

All ICU admissions in 2011 were listed retrospectively. Notes were reviewed for those with blood cultures taken during, or within 24 hours prior to or following, ICU admission. Data collected for positive blood cultures included organism, antimicrobial sensitivity, culture timing with respect to ICU admission and antibiotic therapy before and after positive blood culture. Qualitative decisions were made regarding clinical utility of each positive blood culture. Results were deemed useful if management changed - starting, changing or stopping antibiotics or altering antibiotic duration. Confirmatory results or those not altering treatment were not deemed useful. Statistical analysis was performed using the chi-squared test.

## Results

During 2011 and 2012, there were 450 ICU admissions. In total, 698 blood cultures were taken during, or within 24 hours prior to or following, ICU admission. A total of 135 blood cultures were taken in the 24 hours prior to ICU admission. Of these, 26 grew significant organisms (19.3%). Nine of these cultures were deemed clinically useful (6.7%). A total of 542 blood cultures were taken during ICU admission. Thirty-three of these yielded significant results (6.1%) but only nine (1.7%) were deemed clinically useful. A total of 102 cultures were taken in the first 24 hours of the iCu, of which 17 were positive (16.7%) and six were useful (5.9%). Fifty-three were taken over the next 24 hours, of which two were positive and two were useful (3.8%). Forty-four cultures were taken over the following 24 hours, of which two were positive (4.5%) and one was useful (2.3%). A total of 343 cultures were taken subsequent to this in the ICU, of which 12 were positive (3.5%) and three were deemed useful (0.9%). Of 22 blood cultures taken in the 24 hours post ICU, one was positive but deemed useful (4.5% yield).

## Conclusion

These results demonstrate overall low clinical utility of blood cultures but specifically that utility of ICU cultures is significantly lower than pre ICU (1.7% vs. 6.7%; *P *= 0.0001). The yield of positive blood cultures and their clinical utility also decrease during ICU stay. This may reflect appropriate empirical antibiotics and lower bacteraemia burden in later illness. Given the low clinical yield and a lack of established sensitive or specific triggers [1], we suggest further work in an ICU setting to maximise utility whilst minimising harm.
